# High intensity training improves cardiac function in healthy rats

**DOI:** 10.1038/s41598-019-42023-1

**Published:** 2019-04-04

**Authors:** Maxim Verboven, Anne Cuypers, Dorien Deluyker, Ivo Lambrichts, Bert O. Eijnde, Dominique Hansen, Virginie Bito

**Affiliations:** 1 Biomedical Research Institute, Hasselt University, Hasselt, Belgium; 2 Heart Centre Hasselt, Jessa hospital, Stadsomvaart 11, 3500 Hasselt, Belgium

## Abstract

Exercise training is a low cost and safe approach for reducing the risk of cardiovascular disease development. Currently, moderate-intensity training (MIT) is the most preferred exercise type. However, high-intensity interval training (HIIT) is gaining interest especially among athletes and healthy individuals. In this study, we examined cardiac remodeling resulting from MIT and HIIT in healthy rats. Healthy male Sprague-Dawley rats were randomly assigned to MIT or HIIT for 13 weeks. Animals kept sedentary (SED) were used as control. Cardiac function was evaluated with echocardiography and hemodynamic measurements. Heart tissue was stained for capillary density and fibrosis. After 13 weeks of training, only HIIT induced beneficial cardiac hypertrophy. Overall global cardiac parameters (such as ejection fraction, cardiac output and volumes) were improved similarly between both training modalities. At tissue level, collagen content was significantly and similarly reduced in both exercise groups. Finally, only HIIT increased significantly capillary density. Our data indicate that even if very different in design, HIIT and MIT appear to be equally effective in improving cardiac function in healthy rats. Furthermore, HIIT provides additional benefits through improved capillary density and should therefore be considered as a preferred training modality for athletes and for patients.

## Introduction

Exercise training intervention is recognized as an important and low-cost and safe strategy to prevent and treat cardiovascular diseases^1,2^. Exercising on a regular basis is indeed associated with a decreased risk for coronary artery diseases and heart failure through a reduced blood pressure, increased myocardial perfusion capacity and improved cellular metabolism in the healthy heart^3,4^. Overall, physiological left ventricular (LV) remodeling, as generated by repeated bouts of endurance exercise, has been shown to improve cardiac performance in healthy subjects and increase tolerance for ischemia and reperfusion injury, resulting in a beneficial effect on disease progression and survival in patients with LV dysfunction^5–8^.

In that context, many studies have demonstrated that moderate-intensity endurance training (MIT) is beneficial for cardiac function, both in healthy and pathological situations^8,9^. Recently, high-intensity interval exercise training (HIIT) has raised much attention, not only to assist coaches in adjusting training programs for elite and recreational athletes^10^, but also as a new approach to handle heart failure patients^11,12^. Indeed, HIIT is a widely used and effective training method in various sports, including endurance and sprint events, combining respectively glycolytic and oxidative metabolism^13^. Recent data indicate that HIIT provides indeed additional benefits and could be even more potent than MIT in improving cardiorespiratory function, metabolic response and cardiac function in healthy adults^14–18^. A number of studies attribute this potential to a higher cardiomyocyte mitochondrial fatty acid oxidation and metabolism^15–19^. Data also suggest that HIIT might provide additive benefits compared with the classical MIT modality^19^. In cardiac rehabilitation in T2DM patients, HIIT provides improved systolic blood pressure (SBP), HbA_1C_, high-density lipoprotein cholesterol (HDL-C), malondialdehyde (MDA) levels, Nitric Oxide (NO) and von Willebrand factor (vWF). Overall, HIIT seems superior compared with MIT in improving vascular functions^20^. Importantly, molecular pathways and effects of these different exercise modalities on cardiovascular effects are however still equivocal and remain to be elucidated.

In this study, we hypothesized that HIIT could provide higher cardiovascular gain in physiological remodeling, potentially through improved mitochondrial metabolism and tissue vascularization. The aim of the current study was therefore to perform a thorough comparison on the beneficial effects induced by either MIT or HIIT on cardiac function in healthy rats. This could lead to optimization of exercise programming for healthy individuals, athletes and heart failure patients seeking for strategies to enhance cardiac function.

## Results

### Different exercise types improve cardiac function to the same extent

At baseline, no difference were observed neither in body weight nor in cardiac function assessed by conventional echocardiography between the three groups (data not shown). Thirteen weeks after the start of exercise intervention, sedentary animals gained significantly more weight than trained animals (body weight SED: 596 ± 16 g; MIT: 483 ± 18 g; HIIT: 472 ± 13 g, p < 0.05). Body weight change was comparable between the two training groups.

Conventional echocardiographic characteristics of the animals are summarized in Table 1. Ejection fraction, a parameter for global cardiac function, was significantly improved after 13 weeks of exercise training (p < 0.05). Furthermore, animals undergoing HIIT specifically displayed changes in LV morphology with wall hypertrophy (increased of 24% for AWT and 17% for PWT compared to SED, p < 0.05) while MIT did not induce wall hypertrophy. Finally, EDV was not different between groups, while ESV significantly and progressively decreased with MIT and HIIT intervention (p < 0.05). These changes resulted in a significant increase in SV of 27% in both training groups (p < 0.05), while CO was preserved.

**Table 1:** Conventional echocardiographic parameters after 13 weeks of exercise training.

	SED (N = 10)	MIT (N = 8)	HIIT (N = 8)
HR (bpm)	347 ± 7	325 ± 10	314 ± 13
AWT (mm)	1.4 ± 0.1	1.5 ± 0.1	1.7 ± 0.4*
PWT (mm)	1.4 ± 0.1	1.4 ± 0.1	1.6 ± 0.1*
EDV (µL)	413 ± 24	436 ± 27	426 ± 12
ESV (µL)	154 ± 10	107 ± 10*	95 ± 7*
SV (µL)	259 ± 17	329 ± 22*	331 ± 7*
CO (ml/min)	89.4 ± 5.5	106.7 ± 7.6	104.0 ± 5.7
EF	63 ± 1	76 ± 2*	78 ± 1*

HR, heart rate; AWT, anterior wall thickness; PWT, posterior wall thickness; EDV, end-diastolic volume; ESV, end-systolic volume; SV, stroke volume; CO, cardiac output; EF, ejection fraction. Data are shown as mean ± SEM. *Denotes p < 0.05 *vs*. SED.

Hemodynamic measurements are summarized in Table 2. Both exercise modalities were equally able to decrease LVP as compared to SED animals (p < 0.05). Contractility, as evaluated with dP/dt_max_, as well as relaxation parameters, *i.e*. dP/dt_min_ and tau, were comparable in all groups (p < 0.05).

**Table 2:** Hemodynamic parameters after 13 weeks of exercise training.

	SED (N = 10)	MIT (N = 8)	HIIT (N = 8)
LVP (mmHg)	108 ± 3	96 ± 2*	96 ± 2*
EDP (mmHg)	5.2 ± 0.4	3.9 ± 0.4	3.7 ± 0.6
Tau (ms)	0.01 ± 0.001	0.01 ± 0.001	0.008 ± 0.002
dP/dt_max_ (mmHg/s)	7354 ± 274	7382 ± 277	7340 ± 258
dP/dt_min_ (mmHg/s)	−7530 ± 211	−7489 ± 215	−7190 ± 203

LVP, left ventricular pressure; EDP, end diastolic pressure; Tau, relaxation time constant; dP/dt_max_, peak rate of pressure rise; dP/dt_min_, peak rate of pressure decline. Data are shown as mean ± SEM. *Denotes p < 0.05 *vs*. SED.

Overall, data indicate beneficial cardiac remodeling following exercise training, most global improvements are independent of the exercise modality.

### As opposed to MIT, HIIT training is able to increase capillary density

**Figure 1:**
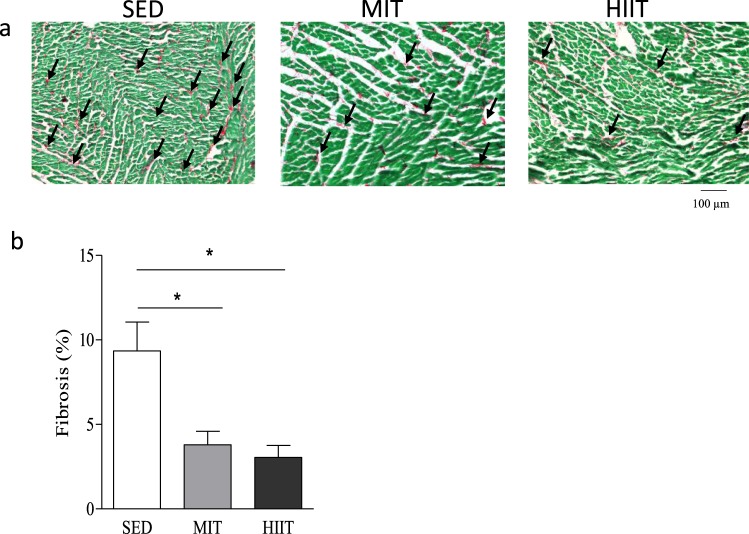
Exercise intervention reduces cardiac collagen content. Representative images of interstitial collagen, as indicated by the arrows, in LV sections obtained with Sirius red/Fast Green in SED, MIT and HIIT groups. (**b**) Total interstitial collagen quantification in SED (N = 10), MIT (N = 8) and HIIT (N = 8). Data are shown as mean ± SEM. *Denotes p < 0.05 *vs*. SED.

**Figure 2:**
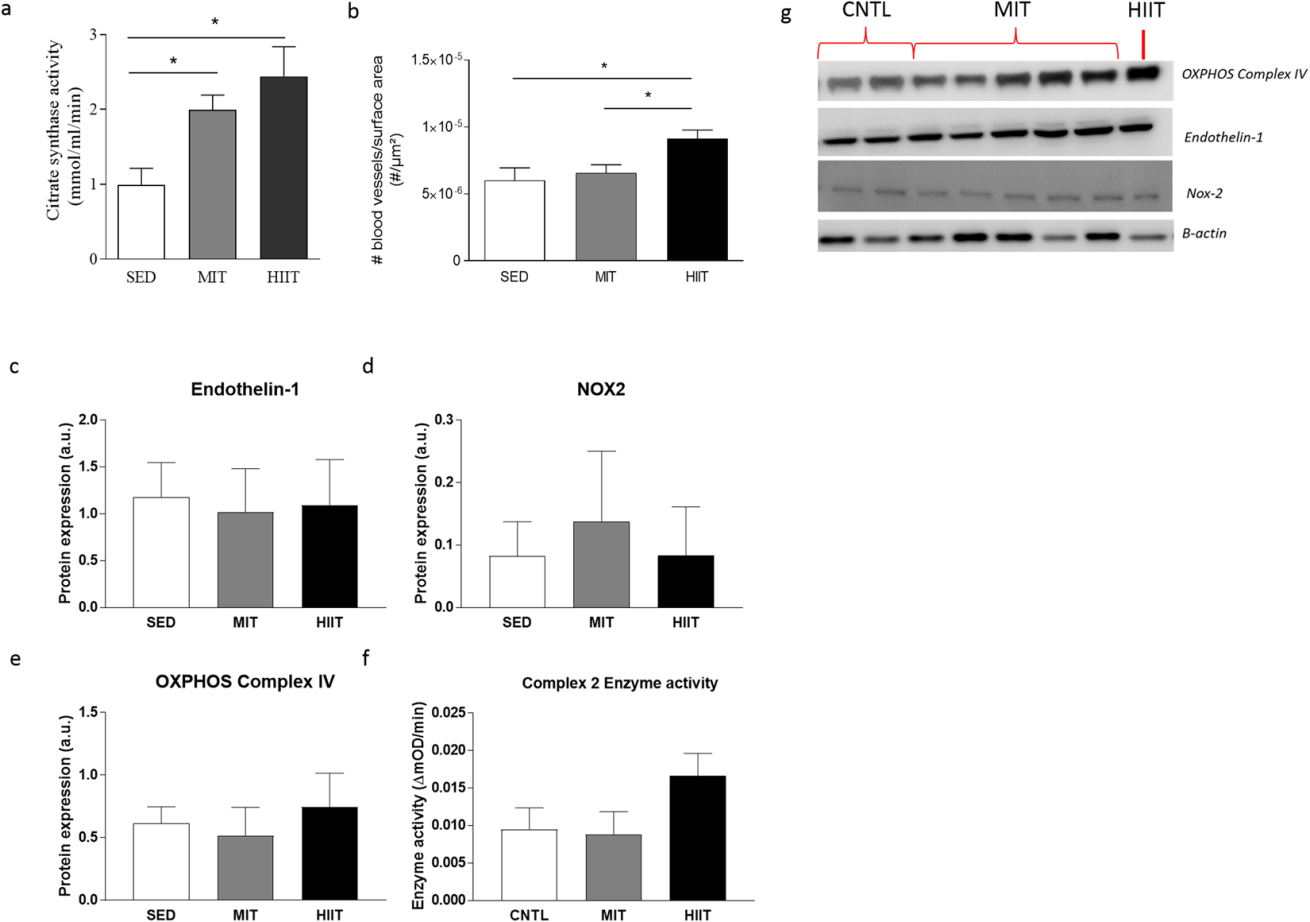
Exercise intervention increases citrate synthase activity and capillary density. (**a**) Citrate synthase activity, measured in LV homogenates, in all groups. (**b**) Capillary density expressed as blood vessels per surface area, in the three groups. (**c**) Protein level of endothelin-1, normalized to β-actin. (**d**) Protein level of NADPH Oxidase 2 (NOX2), normalized to β-actin. (**e**) Protein level of oxidative phosphorylation (OXPHOS) complex IV, normalized to β- actin. (**f**) Enzyme activity of complex II in cardiac tissue. (**g**) Results of representative Western blots. Cropped images of Western blot, for full blots see Supplementary Figs 1–3. All blots were performed individually. Data are shown as mean ± SEM from SED (N = 10), MIT (N = 8) and HIIT (N = 8) for graphs, blots represent a partial blot. *Denotes p < 0.05 *vs*. SED.

Figure 1a are representative images of interstitial collagen obtained with Sirius red/Fast Green in heart sections after 13 weeks of exercise training. As summarized in Fig. 1b, total interstitial collagen was significantly and equally decreased in animals subjected to exercise training. Citrate synthase activity, a marker of aerobic capacity and mitochondrial mass, improved equally in both groups undergoing a training program (p < 0.05) (Fig. 2a). Additionally, capillary density, evaluated by a CD31 staining in cardiac tissue sections, was unchanged with MIT (p > 0.05) but was significantly increased with HIIT (p < 0.05) (Fig. 2b). Finally, no difference was observed in Endothelin-1 (Fig. 2d), NADPH Oxidase 2 (NOX2) nor oxidative phosphorylation (OXPHOS) protein levels (Fig. 2e). Complex II enzyme activity levels were not altered after exercise, however, a positive trend was observed in HIIT animals (Fig. 2f).

## Discussion

In this study, we demonstrate that both MIT and HIIT intervention, although very different training types, lead to beneficial effects on cardiac function in healthy animals. Importantly, HIIT might provide additional benefits as opposed to MIT as HIIT is able to increase blood capillary density, an important feature for optimal cardiac muscle metabolism.

### Both HIIT and MIT lead to beneficial cardiac remodeling, resulting in an improved cardiac function through reduced afterload

While some studies demonstrate that MIT improves cardiac function, others report very little changes^21^. The beneficial effects of exercise training are believed to be mainly attributed to improved neurohumoral, inflammatory, metabolic and central hemodynamic responses as well as on endothelial, skeletal and cardiovascular function, leading to an overall cardiac improvement and an improved tolerance for ischemia and reperfusion injury^5,6^. One major hallmark of LV remodeling related to endurance exercise training, independent of the applied training modality, is the increase in LV mass. Cardiac hypertrophy is commonly accepted to be physiological and not associated with adverse effects, as opposed to pathological hypertrophy which involved substantially different signaling pathways^22–25^. Pathological hypertrophy is related with an increase in collagen accumulation and wall thickness, causing reduced cardiac function^2^ which was not observed here. In our study, we have shown that unlike MIT, HIIT increases AWT and PWT, markers of LV wall hypertrophy, as also confirmed in the study of Hafstad *et al*.^2,26^. In the study of Kemi *et al*. a HIIT modality was more efficient in inducing LV hypertrophy compared when moderate-intensity training was applied^27^. These data suggest that HIIT might be more efficient than MIT in physiological cardiac remodeling. However, whether this is related to activation of different/specific signaling pathways remains to be elucidated. Endothelin-1 is known to promote hypertrophic remodeling, but no change was observed in the different groups in our study, indicating alternative pathways for remodeling after exercise.

Physiological adaptation of cardiac function to exercise training involves structural and metabolic remodeling, resulting in a lower resting HR, improved CO and changed volumes^28^. In our study, independent of the applied training modality, HR and EDV were not statistically different from sedentary animals. Noteworthy, EF, a marker of global cardiac function, was significantly improved with both exercise training interventions. As also shown by Dawes *et al*.^28^, the improved global cardiac function was associated with an increased SV in MIT and HIIT groups. The increase in SV, together with unchanged EDV but reduced ESV, indicates an increased cardiac contractility and/or reduced afterload in trained animals^29^. The effect of endurance training on cardiac contractility remains however controversial. While some studies demonstrate an increased contractility attributed to an improved oxygen delivery, angiogenesis and nitric oxide (NO) sensitivity after endurance exercise training^24,30^, others report very little changes^21^. In our study, dP/dt_max_, a parameter reflecting cardiac contractility, was not changed, indicating that the beneficial cardiac changes observed are likely to be attributed to a decreased afterload, rather than a major change in contractility. This is further confirmed by the decreased LVP, which, combined with a CO that tended to be higher, indicates a decrease in vascular resistance, resulting in a reduced afterload. In that context, the effect of exercise training on endothelial function and, as a result, on vasodilatation, was already well described by others in humans and experimental models^12,31–33^. Indeed, exercise training has been shown to lead to vascular adaptation and improved vascular function through increased NO levels^34,35^. Our *in vivo* data indicate that an improved cardiac function is likely attributed to vascular adaptations, leading to a reduced afterload. However, the exact mechanisms and further study on endothelial function, examining the effect of the different training interventions on NO levels remains to be performed.

It has been shown in rodents that exercise training is not necessarily accompanied by myocardial collagen change^24^. In our study, we show that both exercise interventions were able to substantially decrease interstitial collagen content in cardiac muscle. This decrease in fibrosis was however not strongly associated with improvements of diastolic function as previously described^36–39^. Indeed, despite the unchanged EDV and unchanged CO, EDP only tended to be decreased in both training modalities (p = 0.07). Whether an improved diastolic function related to a decreased fibrosis content following exercise training can be completely ruled out remains to be further confirmed as it has been shown that diastolic function (*i.e*. EDP) is directly associated with cardiac fibrosis level^40,41^. However, most experiments examining fibrosis and diastolic function have been performed under pathological settings^39,42^. In our study, we examined physiological remodeling in healthy rats. Therefore, the effect of exercise on cardiac fibrosis level and its impact on diastolic function is likely to be substantially more important in pathological situations as levels of fibrosis in pathological settings are at least 2 times larger than what is reported in healthy situations^40,43^.

### What is the added value of HIIT?

It is known that an increase in cardiac capillary density is important for the development of physiological hypertrophy^44,45^. In that context, the link between angiogenesis and hypertrophy has been previously emphasized^44^ where the seen effects were shown to be mediated through paracrine effects and secretion of NO and growth factors (such as VGEF, NRG-1). In our study, only HIIT was able to increase capillary density, indicating a somehow higher potential for HIIT as compared to MIT. Very recently, further confirming our results, the study of Machado *et al*. demonstrated that exercise training was able to prevent capillary rarefaction in obese rats with the metabolic syndrome^46^. An adequate capillary density allows for greater oxygen transport to the cardiac muscle and improves ultimately cardiac metabolism. In our study, citrate synthase activity, a measure of mitochondrial mass, was significantly improved after MIT and HIIT. Together with this, complex II enzyme activity seemed to be increased in HIIT compared with sedentary animals, however not significantly. Our data therefore suggest that HIIT might provide additional benefits compared to MIT, through increased capillary density and the resulting improved oxidative metabolism, not limited to the skeletal muscle as shown by Takada *et al*.^47^ but also in the heart, as suggested in the current study and by others^48^. Cardiac metabolism requires aerobic production of ATP. In our study, HIIT provides increased cardiac vascularity to counter high oxygen requirements during exercise, as in HIIT exercise is performed at almost maximal oxygen consumption. Increased mitochondrial activity and mass will result in an increased ATP production, necessary to keep up with the cardiac energy demand^49^. Since blood supply is essential to improve cardiac function, particularly in pathological situations where blood vessels are dramatically reduced^50^ or when angiogenesis needs to be restored to optimize cardiac repair through stem-cell therapy for instance^51^, HIIT might be a great advantage compared to MIT. However, further investigation is required to draw conclusions as underlying changes related to the different training interventions on endothelial function, including mitochondrial morphology and mitochondrial function, are so far unknown. Exercise and oxidative stress have a complicated relationship, as exercise can have both a positive and negative effect on oxidative stress^52^. NOX-2 plays a role in hypertrophy and interstitial fibrosis, is a super-oxide generating enzyme playing a role in oxidative stress and reactive oxygen species (ROS)^53^. However, in our study, no effect on oxidative stress levels was observed after exercise. This can be due to multiple causes, as mode, intensity and duration of the exercise are known to play an important role in the observed result.

Finally, because training modalities used in HIIT are short in time, motivation and adherence of patients to HIIT training as compared to MIT, could be potentially higher. This could be a serious advantage in the clinical setting since both trainings offer beneficial cardiac remodeling with an added value to HIIT at the molecular level. Last but not least, not limited to a pathological setting, one could speculate that HIIT could be a way to improve in a more efficient and faster way, cardiovascular function in athletes. However, translation of results from bench to bed-side should be done with caution taking into account the differences between animal and human studies.

## Conclusion

Our data indicate that even if very different in design, HIIT and MIT appear to be equally effective in improving cardiac function in healthy rats. Furthermore, HIIT shows some promising results and might provide additional benefits through improved capillary density. Therefore, it could be considered as a preferred training modality for athletes and for patients for whom improvements in cardiac function are aimed at.

## Methods

This investigation conforms to the EU directive 2010/63/EU for animal experiments and was approved by a local ethical committee (Ethische Commissie Dierproeven, UHasselt, Diepenbeek, Belgium). All experiments and methods were performed according the relevant guidelines.

### Study protocol and exercise intervention

Twenty-six male Sprague-Dawley rats (Charles River Laboratories, L’Arbresle, France), weighing 200–225 g were used throughout the study. All animals were familiarized with the treadmill (IITC, California, United States of America) prior the experiment (for 3 days/week) and were randomly assigned (by randomization program) to one of the three experimental groups after 2 weeks familiarization. Group 1 did not undergo exercise training throughout the study (SED, N = 10). Group 2 was subjected to moderate intense endurance training schedule, consisting in running on a treadmill, 18 m/min, 5° inclination, 1 h/day, 5 days/week (MIT, N = 8). Group 3 consisted in 10 bouts of high-intensity treadmill running (18 m/min, 30° inclination), separated by 1 min of active rest, 5 days/week (HIIT, N = 8). Sample size was calculated by a Power analysis based on previous results.

The intensity of exercise training was assessed by measuring blood lactate levels directly after exercise with an Analox apparatus (Analis, Namur, Belgium). Levels > 4 mmol/l lactate were considered HIIT^54^. Training modalities were adjusted to lead to an equal energy expenditure between interventions by calculating the net caloric cost (kcal/min) using following calculations:

- VO_2_ max = S_a_ * 0.2 + (S * G_b_) * 0.9 (S_a_ = speed (m/min), S = speed (m/min), G_b_ = inclination);
- Net caloric cost (kcal/min) = VO_2_ max * 3.5 * body mass (kg)/200

All animals were maintained in a controlled environmental condition of temperature and humidity and had water and food (normal rodent diet, ENVIGO, The Netherlands) available *ad libidum*. Blood samples, hemodynamic measurements and echocardiographic measurements were executed 13 weeks after the start of the training program.

### Conventional echocardiographic measurements

Prior to sacrifice, transthoracic echocardiography was performed under 2% isoflurane in all animals with a Vivid I ultrasound machine (GE Vingmed Ultrasound) using a 10 MHz linear array transducer. The protocol used is as described previously^40,41^. Briefly, a standard parasternal long axis image and short axis views at the mid-ventricular level were obtained at a temporal resolution of approximately 200 frames per second. Conventional echocardiographic parameters (*e.g*. LV end-diastolic diameter (LVEDD), LV end-systolic diameter (LVESD), posterior wall thicknesses (PWT) and anterior wall thicknesses (AWT)) were obtained from the B-mode images at midpapillary level in the parasternal short-axis view. End-systolic volumes (ESV) and end-diastolic volumes (EDV) were calculated by π*DM2*B/6, where DM indicates the systolic/diastolic diameter of the ventricle in mid-ventricular short-axis view and B is LV length on parasternal long-axis image. Subsequently, ejection fraction (EF) was measured as (EDV–ESV)/EDV, and expressed in %.

### Hemodynamic measurements

Conductance measurements were performed in all animals with the use of an SPR-320 MikroTip high-fidelity pressure transducer (Millar Inc) that was advanced into the left ventricle via the right carotid artery, as described previously^41^. The pressure catheter (2 F) was connected to a quad-bridge amplifier and PowerLab 26 T module (AD Instruments, United Kingdom) was used to transfer the data to LabChart v7.3.7 software (AD Instruments, United Kingdom).

### Citrate synthase activity in cardiac homogenates

Citrate synthase activity was determined in LV cardiac homogenates using a citrate synthase assay kit (CS0720; Sigma-Aldrich, St. Louis, MO)^55^.

### Fibrosis and capillary density measurements in tissue sections

Transversal sections of 7 µm thick were obtained at cardiac midventricular level and stained using the Sirius Red/Fast Green kit (Chondrex). Fibrosis was assessed in all animals in four randomly chosen fields per section. The area of collagen deposition indicated by red staining was outlined and quantified using an automated image analysis program (Carl Zeiss, AxoVision 4.6, Zaventem, Belgium). Blood vessels were excluded. Total collagen deposition to the global cardiac area was calculated and expressed as percent collagen deposit.

Capillary density was quantified from histological sections by immunohistochemical staining for CD31 (SC-1506, Santa Cruz, 1/100). Capillaries were visualized by 3-3-diaminobezedine (DAB) and counterstained using hematoxylin. The amount of blood vessels were counted in 10 different fields per section and averaged. Data are expressed as amount of capillaries per µm^2^.

### Endothelin-1, OXPHOS and NOX2 protein levels

Protein concentrations of the LV tissues were determined by the BCA protein assay kit (Thermo Fisher, Erembodegem, Belgium). Western blot was performed as previously described^40^. Briefly, equal amounts of proteins (15 µg) were separated on a 12% SDS-PAGE gel with a mini protean 3 electrophoresis system (Bio-rad Laboratories, Temse, Belgium), transferred to a polyvinylidene fluoride (PVDF) membrane and subsequently, blocked for 2 h with 5% milk in Tris-buffered solution containing 0.1% Tween-20 (TBS-T) followed by incubation overnight at 4 °C in the presence of a specific endothelin-1 antibody (1/2500, Abcam, ab117757, Cambridge, United Kingdom), NOX2 antibody (1/2500, Abcam, ab31092, Cambridge, United Kingdom) or an OXPHOS antibody (1/1000, Abcam, ab110411, Cambridge, United Kingdom). Horseradish peroxidase-conjugated secondary antibodies (DAKO, Belgium) at a dilution of 1/2000 were used. Both primary and secondary antibodies were diluted in 5% milk-TBS-T. Visualization was performed with the enhanced chemiluminescence (ECL) technique using the Pierce ECL Plus western Blotting Substrate (Thermo Fisher, Erembodegem, Belgium). Data were normalized to β-actin protein levels.

### Complex II enzyme activity

Complex II enzyme activity in cardiac tissue was determined using a Complex II Enzyme Activity Microplate Assay Kit (Abcam, ab109908, Cambridge, United Kingdom). Samples were prepared as described in the protocol, and added to an anti-Complex II monoclonal antibody-coated 96-well plate. Absorbance was measured at OD600 nm for 60 minutes, measuring every 20 seconds.

### Statistical analysis

Results were tested for normality prior to statistical tests. Subsequently, one-way ANOVA test was performed combined with a post- hoc test, dependent of normality. If data were normally distributed, Tukey’s range test was performed. Elsewise, the Dunn’s test was used. Analyses were performed using GraphPad Prism (GraphPad Software, San Diego, CA, USA). A value of p < 0.05 was considered statistically significant.

## Supplementary information

Supplementary Dataset 1
