# Supplementary material for: High intensity training improves cardiac function in healthy rats
Source: Sci Rep. 2019 Apr 4;9:5612. doi: 10.1038/s41598-019-42023-1 (PMC6449502; doi:10.1038/s41598-019-42023-1)
Supplement: Supplementary file 1 — Supplementary Dataset 1 [file 41598_2019_42023_MOESM1_ESM.pdf]

High intensity training improves cardiac function in healthy rats

Maxim Verboven[1], Anne Cuypers[1], Dorien Deluyker[1], Ivo Lambrichts[1], Bert O Eijnde[1], Dominique Hansen[1,2]~ Virginie Bito[1]*~

~ Equally contributing

[1] Biomedical Research Institute, Hasselt University, Belgium

[2] Heart Centre Hasselt, Jessa hospital, Stadsomvaart 11, 3500 Hasselt.

***Address for correspondence**:

Virginie Bito

Biomedical Research Institute (BIOMED)

University Hasselt

Martelarenlaan 42

BE 3500 Hasselt

Virginie.Bito@uhasselt.be

Tel: +32-11 26 92 85

Fax: +32-11 26 92 99

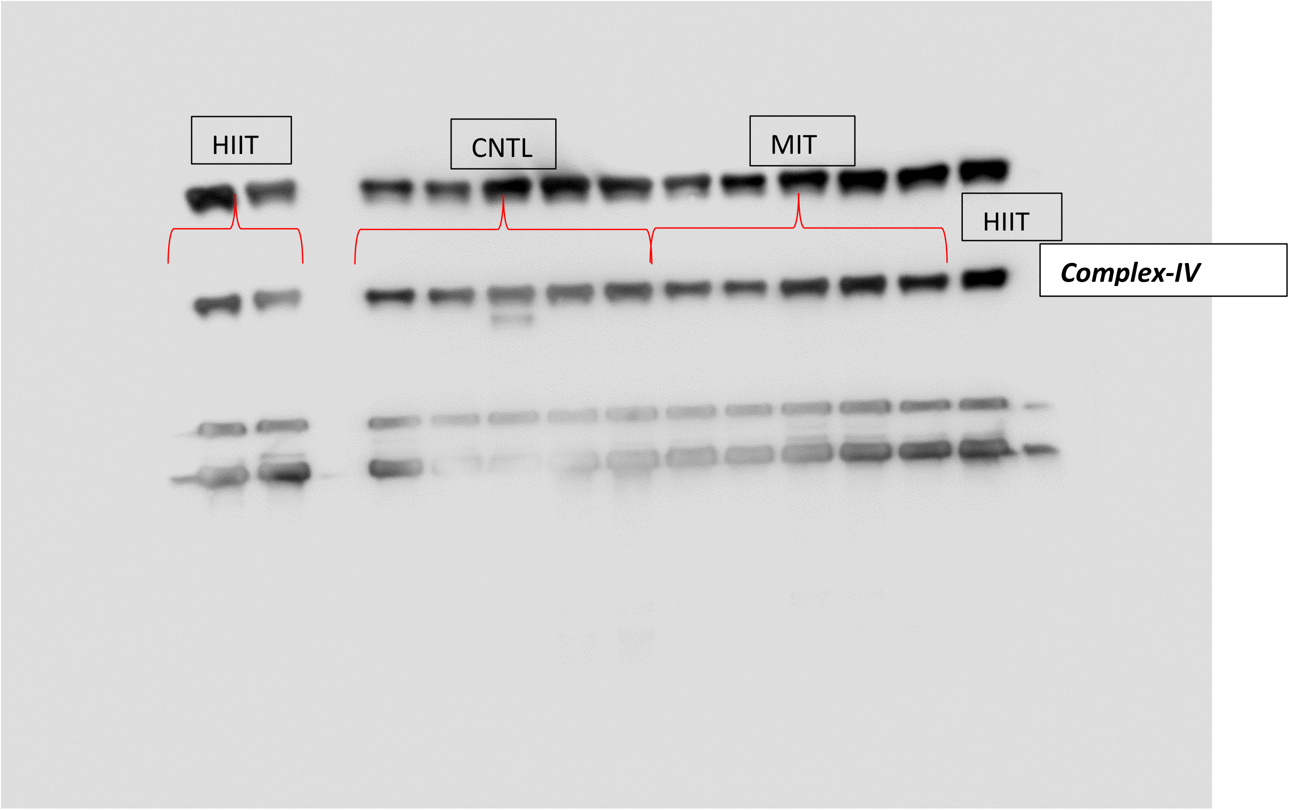

Figure 1: Representative Western blot of OXPHOS antibody.

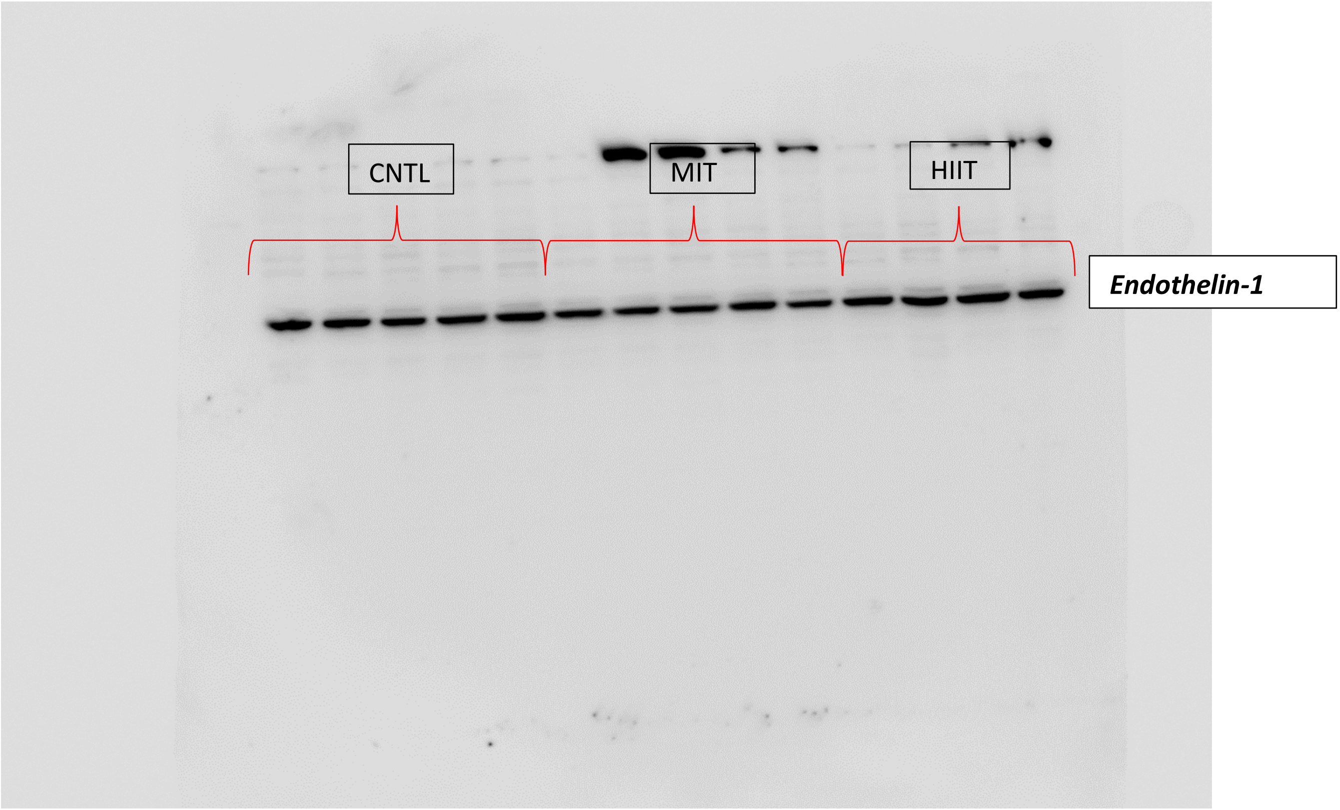

Figure 2: Representative Western blot of Endothelin-1 antibody.

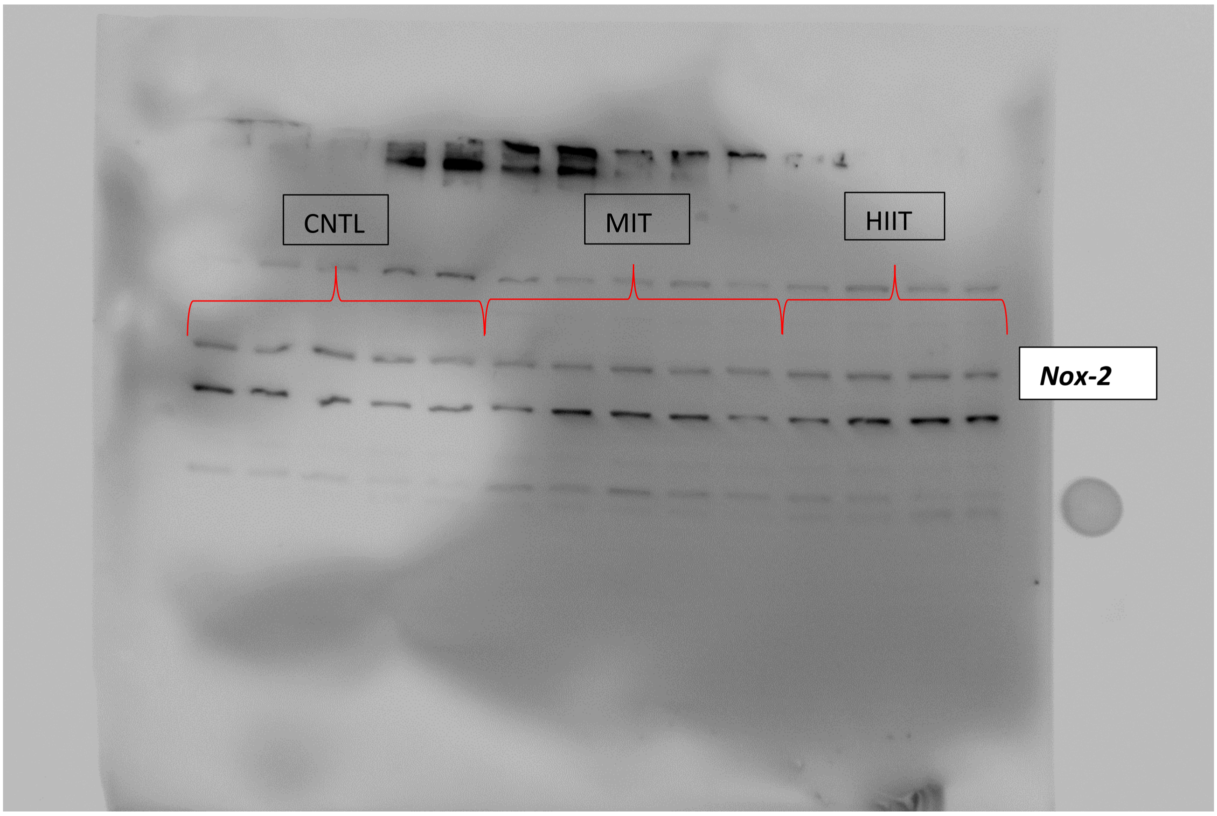

Figure 3: Representative Western blot of Nox-2 antibody.
